# Observational and Longitudinal Study on the Relationship Between the Transition Pathway in Gender Dysphoria and the Presence of Comorbid Anxiety and Depressive Symptoms

**DOI:** 10.1192/j.eurpsy.2026.11920

**Published:** 2026-07-31

**Authors:** A. Zendoli, R. Ferrara, A. bellomo

**Affiliations:** 1 Department of Clinical and Experimental Medicine, University of Foggia, Italy, Ospedali riuniti, Foggia; 2 ASL FG, Lucera; 3 department of Clinical and Experimental Medicine, University of Foggia, Italy, Università di Foggia, Foggia, Italy

## Abstract

**Introduction:**

Gender dysphoria (GD) is frequently accompanied by anxiety and depressive symptoms. This real-world longitudinal observational study examined the evolution of these symptoms during the early stages of gender affirmation.

**Objectives:**

To assess changes in depression and anxiety from T0 (intake) to T1 (~1 month after GD diagnosis) and T2 (6 months after initiation of gender-affirming hormone therapy); (2) to compare clinician-rated (HDRS/HAM-D; HARS/HAM-A) and self-report measures (BDI-II; STAI-Y1/Y2); (3) to explore T0–T1 correlations; and (4) to describe socio-clinical features and psychotropic medication use.

**Methods:**

Seventeen adults were followed at an outpatient GD clinic (mean age = 25.5 ± 6.7 years; 58.8% assigned female at birth; 10 FtM, 7 MtF). RMANOVA was performed on nine participants with complete T0–T1–T2 data for each scale, while Pearson correlations were computed for all 17 participants with T0–T1 data. Clinical, demographic, and treatment information were collected.

**Results:**

Repeated-measures ANOVA indicated significant improvements over time for HDRS (F(2,16) = 3.80; p = 0.045), HAM-A (F(2,16) = 4.77; p = 0.024), BDI-II (F(2,16) = 42.58; p < 0.000001), STAI-Y1 (F(2,16) = 16.47; p = 0.00013), and STAI-Y2 (F(2,16) = 15.00; p = 0.00021). Trajectories were more homogeneous for self-report measures, with marked reductions already evident at T1 and further declines at T2. T0–T1 correlations were weak/non-significant for HDRS (r = 0.36; p = 0.152) and HAM-A (r = 0.44; p = 0.081), but strong and significant for BDI-II (r = 0.89; p = 0.000002), STAI-Y1 (r = 0.87; p = 0.000006), and STAI-Y2 (r = 0.84; p = 0.000023).

**Conclusions:**

Access to dedicated care and initiation of the gender affirmation process—including hormone therapy—were associated with clinically and statistically significant reductions in depressive and anxiety symptoms, more consistently detected by self-report instruments. Clinician-rated scales reflected greater inter-individual variability, suggesting heterogeneous trajectories of change. Despite the small sample and naturalistic design, these findings support integrated, culturally sensitive, multidisciplinary care and highlight the need for larger longitudinal studies.

**Disclosure of Interest:**

None Declared

